# Fused Filament Fabrication for Metallic Materials: A Brief Review

**DOI:** 10.3390/ma16247505

**Published:** 2023-12-05

**Authors:** Jose M. Costa, Elsa W. Sequeiros, Manuel F. Vieira

**Affiliations:** 1 Department of Metallurgical and Materials Engineering, Faculty of Engineering, University of Porto, R. Dr. Roberto Frias, 4200-465 Porto, Portugal; ews@fe.up.pt (E.W.S.); mvieira@fe.up.pt (M.F.V.); 2 LAETA/INEGI—Institute of Science and Innovation in Mechanical and Industrial Engineering, R. Dr. Roberto Frias, 4200-465 Porto, Portugal

**Keywords:** additive manufacturing, solid-state processes, material extrusion, fused filament fabrication, metallic materials

## Abstract

Fused filament fabrication (FFF) is an extrusion-based additive manufacturing (AM) technology mostly used to produce thermoplastic parts. However, producing metallic or ceramic parts by FFF is also a sintered-based AM process. FFF for metallic parts can be divided into five steps: (1) raw material selection and feedstock mixture (including palletization), (2) filament production (extrusion), (3) production of AM components using the filament extrusion process, (4) debinding, and (5) sintering. These steps are interrelated, where the parameters interact with the others and have a key role in the integrity and quality of the final metallic parts. FFF can produce high-accuracy and complex metallic parts, potentially revolutionizing the manufacturing industry and taking AM components to a new level. In the FFF technology for metallic materials, material compatibility, production quality, and cost-effectiveness are the challenges to overcome to make it more competitive compared to other AM technologies, like the laser processes. This review provides a comprehensive overview of the recent developments in FFF for metallic materials, including the metals and binders used, the challenges faced, potential applications, and the impact of FFF on the manufacturing (prototyping and end parts), design freedom, customization, sustainability, supply chain, among others.

## 1. Introduction

Additive manufacturing (AM) processes, commonly called 3D printing, are receiving the attention of several industries. These processes allow layer-by-layer construction of complex and customized shaped parts from engineering materials directly from design, without using expensive tooling [1,2,3,4,5]. To accomplish the AM potential, continued research and development on processes and equipment are essential to enable full manufacturing readiness and understanding of the materials [3,4]. AM processes in general, from laser to solid-state processes, can create complex shapes and components from various materials, including plastics, metals, ceramics, and composites, like metal matrix composites (MMC), functionally graded materials (FGM), high entropy alloys (HEA), and others [6,7,8,9,10]. The beam (laser or electron) powder processes are the most used technologies in metal AM. The pertinence and capabilities of this type of process are widely demonstrated and recognized. Thus, this technology has emerged as challenging, since layer-by-layer manufacturing with a heat source leads to columnar grain formation due to the directionality of heat extraction. This microstructure has characteristics dissimilar from those of traditional processes; it usually reveals an anisotropy that degrades mechanical strength in Z-axis directions (or with enhanced strength in the XY-axis), resulting in components that can have unpredictable mechanical behavior, incompatible with parts that require stringent properties [11,12,13,14]. The metal AM processes require high-end and digital technology, like hardware, software, and procedures. They are an intrinsic part of a new industrial paradigm to increase efficiency and productivity by ensuring sustainability and the improvement of the circular economy [3,9,15,16,17].

Fused filament fabrication (FFF) is a material extrusion (MEX) process where material under a filament form is selectively dispensed through a nozzle [18], and it is grouped inside the additive manufacturing (AM) solid-state processes [2,19], where applying debinding and sintering is required. FFF uses a continuous feed of material or mixing of materials, a mixture of metal powders with polymeric binder systems [11], which are melted and deposited layer-by-layer to build an object [20,21,22,23]. FFF is known for its cost-effectiveness and versatility, and is popular for many applications in the automotive, aerospace, and healthcare industries, as well as consumer and markets in general [11,24,25,26,27], since it allows the user to design, create, develop, and produce almost any product with design freedom, flexibility, and versatility when compared to traditional manufacturing [1,3,4,28].

One of the mandatory requirements to achieve an effective layer joining in AM, independently of the selected technology, is to have a proper combination of the feedstock (or raw) material and good energy delivery [29]. As mentioned before, in the FFF process, a filament composed of a binder system mixed with metal powder particles, which is fed through a nozzle, is used to produce components according to the 3D model, and layered according to it; in the end, a green part is achieved [26,27,30]. Afterward, the steps of debinding and sintering treatments are required to create fully metallic parts. The debinding is a critical stage in the production of FFF parts, in which the polymeric binder systems that have been instrumental in holding the powders together are carefully and methodically removed, obtaining the densified brown parts. To attain components with desired densities and structural integrity, the brown parts are sintered through controlled heating, resulting in solid and dense structure components [11,31,32,33,34]. 

This paper aims to provide a comprehensive overview of metallic materials FFF, including its principles, advantages, limitations, design considerations, advanced materials utilization, potential applications and prospects, and directions for research and development of metal FFF.

## 2. The FFF Process for Metallic Materials

FFF has gained recognition as a potential substitute for laser or electron beam processes, mainly due to the easy process and easy-to-use equipment, and smaller overall costs and reduced initial investment, creating new opportunities for advancing metal AM technology [1,5,6,35,36]. The flexibility and widespread accessibility of FFF highlight its utility in various domains such as engineering, design, research, and art, allowing for the production of a diverse range of objects, including prototypes, functional components, models, and intricate artistic sculptures [5,27,37]. 

**Figure 1:**
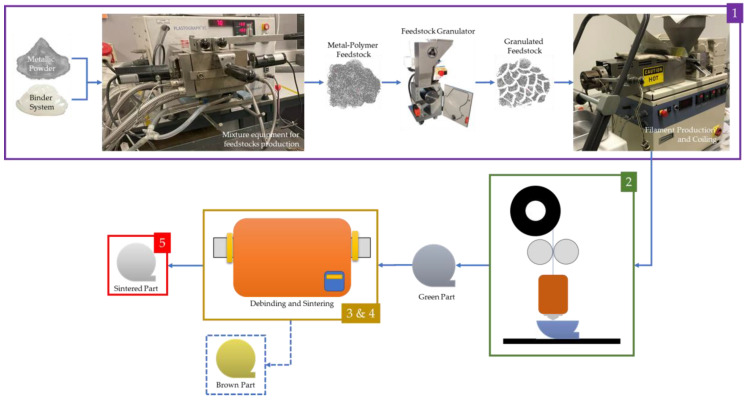
Schematics of FFF for metallic filament feedstock: (1) filament production (including material selection and mixing); (2) production of AM component (green part) using the filament extrusion process; (3) debinding (brown part); (4) sintering, and; (5) sintered part.

FFF for metallic parts can be divided into five steps: (1) raw material selection and feedstock mixture (including palletization), (2) filament production (extrusion), (3) production of AM components using the filament extrusion process, (4) debinding, and (5) sintering. Figure 1 represents the schematics for these five steps of the FFF process.

The production of filaments for metal-based AM is a critical step in the process. It includes material selection and mixture and the extrusion process into the form of the filament. The selection of appropriate materials, including metallic powders, binders, and additives, is essential to producing successful parts. The filaments utilized in metal-based AM result from feedstock extrusion, combining metallic powders and a polymer binder system in specific proportions, typically with 60% metallic powders and 40% polymeric materials. The objective of this mixture, similar to metal hot embossing and metal injection molding, is to distribute the metal powder evenly throughout the binder, preventing internal porosity and agglomeration and resulting in a homogeneous biphasic mixture [3,6,26,38,39,40]. The powder characteristics and properties significantly impact the filaments’ rheological behavior during their manufacture and the FFF process. Ideally, filament characterization should include results such as powder particle size distribution, powders morphology, density, specific surface area, inter-particle interaction, thermo-gravimetric analysis, and differential scanning calorimetry [3,40,41,42,43]. Filament production is a complex operation where the selected process parameters must suit the specific compound and extrusion system used and preliminary optimized for each filament. The filament is usually extruded into a spool. It must be pre-optimized to ensure the blend is homogeneous and has a consistent shape and diameter (usually 1.75 mm or 2.85 mm diameter). A suitable and practical FFF process requires the combination of an appropriate feedstock and extrusion, which enables a stable and effective (and profitable) process [3,27]. The core features for the FFF process in metallic materials are the correct powder load and melt of the filament, adequate pressure to push the molten material through the nozzle to enable a controlled extrusion in the correct coordinate (defined in slicing), and proper bonding of the material extruded, which all create a solid structure [5,26,35,44]. 

There are other processes similar to FFF, which avoid the production of filaments that are being developed. Fused granular fabrication (FGF), where it is possible to use pellets (or granulates) as feedstock [20,21,45,46,47] instead of filament, offers several benefits, including a broader range of material options, lower costs, and reduced waste production. However, using pellets also presents challenges, such as higher temperatures required to melt the pellets, more force needed to push them through the nozzle, and limited availability of specific materials [21,45]. Direct ink writing processes (also known as material jetting (MJT)), where an ink with nanometric metal powder is used as feedstock, where the droplets of the metallic ink are selectively deposited using piezo printing heads. Subsequent curing, often through processes like sintering, solidifies the metal particles, layer-by-layer, to form the desired 3D object. This technology enables the creation of intricate and customized metal components with a high level of precision, making it suitable for various applications, including prototyping and functional part production [48,49,50,51,52,53,54]. However, parts produced with this technology have high porosity and insufficient cohesion between layers [54]. Binder jetting (BJT) builds components by selectively depositing a liquid binder (bonding agent) onto layers of metallic powder to join them. This process operates at ambient temperature, which mitigates thermally induced defects (common in other heat-dependent AM methods), where the surrounding metal powder acts as both a structural component and temporary support, eliminating the need for additional supports and minimizing waste. The process involves depositing a fine layer of metallic powder onto a build platform, followed by the controlled application of binder through an inkjet printhead, resulting in a uniform distribution achieved through capillary pressure and gravitational forces [55,56,57]. Parts produced by binder jetting also have internal porosity, which affects mechanical behavior; in particular, fatigue and fracture resistance can have a very negative impact from this porosity [57,58].

**Figure 2:**
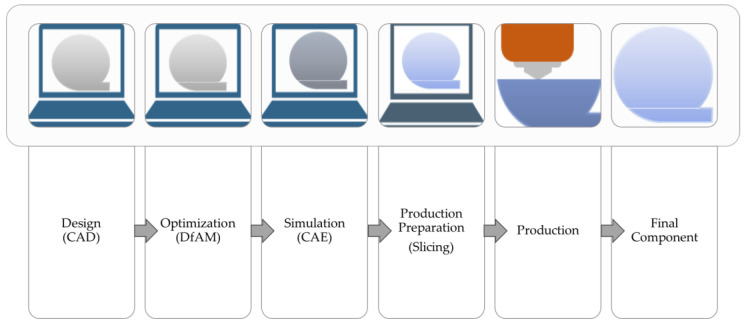
FFF workflow.

In the realm of AM, particularly in FFF (and similar processes), a well-structured workflow is crucial for producing high-quality FFF components, involving several key stages before production [3,4,5], including design (CAD), optimization (design for AM–DfAM), simulation, and production preparation (using slicing software), as seen in Figure 2.

In the FFF process, common to various AM technologies, the initial phase involves designing components using CAD software. Design for AM plays an important role since it explores the unique capabilities and constraints of AM technologies, including lightweighting, complex geometries, and customization, to achieve specific structural and functional objectives while minimizing material usage. The component undergoes a simulation phase through CAE to assess its structural integrity, performance, and other critical factors. Once the 3D CAD model is completed, after design optimization and simulation, the next step is to employ specific software to slice the model, defining the manufacturing strategy, material deposition path, and production parameters (like layer thickness, the number of shells, pattern, raster gap, and speed) [3,21,59,60]. This transformation process, facilitated by slicer software, converts the CAD 3D model into a sequence of paths defined by X, Y, and Z coordinates, forming the foundation for creating the 3D object layer-by-layer. Each slice, essentially a 2D path, guides the nozzle in the manufacturing process to build the component, and collectively, these slices culminate in the 3D object’s formation. Slicers are instrumental in optimizing and parameterizing various properties, profoundly impacting the overall quality and surface finish of the final component [21,44,61,62,63,64]. After slicing, the production phase can be started, fabricating components according to the predefined paths and parameters. 

**Figure 3:**
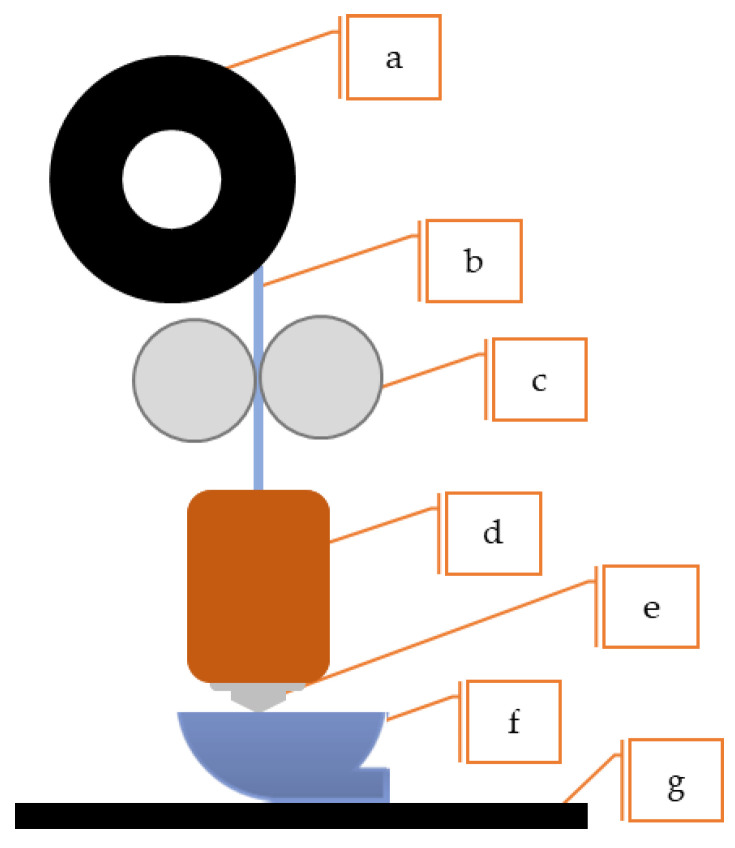
Schematics of FFF equipment: (**a**) metallic spool, (**b**) metallic filament, (**c**) heated chamber, (**d**) extrusion screw, (**e**) nozzle, (**f**) metal AM part, (**g**) build plate.

The FFF equipment (schematics available in Figure 3) for polymer- or metal-based components is similar from an equipment perspective. The feeding mechanism is positioned differently depending on whether the filament is metal- or polymer-based. In the case of filaments constituted by binder systems and metal powder, which are fragile and prone to breakage, the filament spool is usually located at the top of the machine and directly connected to the feed mechanism. To avoid breakage, metallic filaments are fed vertically to the direct extrusion head and are typically used in a temperature-controlled chamber during AM processes. When heated to a temperature range of 150 °C to 200 °C, the filaments acquire a pseudo-plastic state enabling them to be fed into a hot nozzle. The material is then extruded through an orifice diameter ranging from 0.25 mm to 1 mm [1,2,65,66,67]. The material is deposited along the rasterizing paths created in the slicing software. The software determines the deposition of successive layers along a predefined path to create the desired geometry. FFF equipment may feature various heated die nozzles that enable the extrusion of different materials, such as component material, a release layer, or soluble support material [7,28,63,67]. The layer thickness and infill pattern can also be modified to control object strength and weight. The resulting part from this process is called the green part [24,68,69].

**Figure 4:**
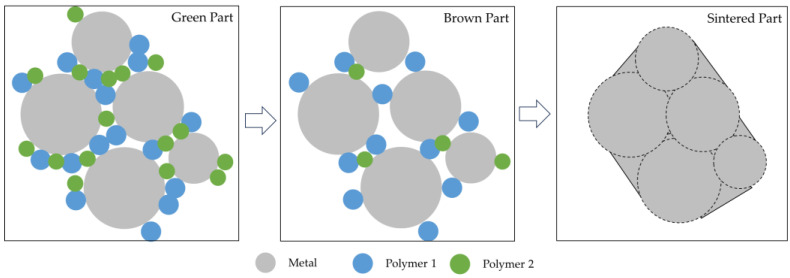
Schematics of debinding and sintering phenomenon.

As mentioned earlier debinding and sintering are required to create fully metallic parts (Figure 4).

Debinding optimization is contingent upon the attributes of the binder system and constituents. Multiple debinding methods are available, such as solvent debinding, catalytic debinding, thermal debinding, or combining two or more techniques [38]. The debinding aims to eliminate the binder systems progressively, keeping the produced components’ shape [3,39]. Obtaining proper components after debinding (brown parts) requires a gradual and stable binder removal to avoid defects and shape loss [6,38]. Poor debinding conditions can influence the components’ porosity since carbon residues (resulting from polymer/binder residues) influence the sintering process, promoting bloating, blistering, surface cracking, and large internal voids, which increases the difficulty of achieving a highly dense component [3,27]. The process of transforming the brown part into a fully dense metal component is sintering, where a heat treatment is applied to transform the loosely bound metal powder particles into a bulk material. The temperature used in sintering is below the melting point (between 70 and 90%) of the metal powder, or the major metallic component, to obtain solid components, with all geometries created in the FFF process [6,27]. In sintering, the combination of high temperature and high porosity of the components, created by removing binders during debinding, promotes intense mass transport. As the sintering temperature increases, the system progressively reduces surface energy, forming solid bonds (or necks) between the metal powder particles; these bonds continue to grow by diffusion, thus, decreasing the porosity and densifying the components, resulting in a shrinkage (linear) of components by around 20% [26,70,71]. To optimize the sintering parameters and enhance component consolidation, it is required to monitor the process through microstructural evaluation and mechanical characterization [3,27].

## 3. Manufacture and Design Considerations for FFF with Metallic Materials

Some specific design considerations must be taken to produce high-quality metallic parts using FFF technology. In fact, compared to the FFF of polymeric materials, applying the process to metallic materials requires special concerns. Metallic materials have different properties than polymers, such as higher melting points, thermal conductivity, and mechanical strength [72]. These properties can affect the part’s production process and final quality. Therefore, selecting a metallic material suitable for FFF and understanding its properties is essential to optimize the manufacturing process [4,23,70]. Another consideration is the capabilities of the FFF equipment. They were designed to produce polymeric materials and may not be suitable for metallic materials [22,35]. It is essential to use equipment specifically designed for producing metallic materials or one that can be modified to produce them [24]. The adhesion to the build plate is also crucial when using metallic materials since they tend to warp more than polymers due to their higher thermal conductivity. Therefore, it is necessary to use a production plate surface that provides good adhesion and prevents warping. The build plates for metallic materials include paper, glass, polyetherimide, and others. The layer height in FFF affects the surface finish and the strength of the finished part. For metallic materials, it is recommended to use a layer height of less than 50% of the nozzle diameter to ensure good adhesion between the layers and avoid delamination [22]. Cooling is also essential since metallic materials require adequate cooling during production to prevent overheating and warping. It is recommended to use equipment with a cooling fan. Finally, after component production, metallic parts produced with FFF may require post-processing to remove support structures, smooth the surface, and improve the part’s mechanical properties. Standard post-processing techniques for metallic parts include sandblasting, polishing, and heat treatment. Regarding supports, some equipment suppliers currently use a ceramic filament to enable an easier separation from parts; the ceramic particles do not sinter due to the higher temperatures required for solid-state diffusion compared to metallic powders [4,5,23]. 

Unlike traditional manufacturing methods, AM technologies build parts layer-by-layer, using CAD data to create complex geometries and intricate internal structures that would be impossible or difficult to produce with conventional manufacturing methods. Regarding design, it does not make sense to design components as if they were produced with the same principles as traditional manufacturing process structures. The customization potential for the FFF of metallic materials significantly influences design considerations [73]. Several methodologies come into play in any AM process to optimize designs. DfAM is essential, focusing on the layer-by-layer approach, overhangs, and print orientation. Topology optimization (TO) and generative design can generate structural sound and innovative designs [74,75,76,77,78,79]. This is complemented with the use of lattice structures, minimization of support structures, and consideration of anisotropy, which are integral to the process [35,80,81,82,83]. Material selection and heat management play fundamental roles, as does using both CAD software tools and iterative prototyping. Simulation and analysis, along with continuous material and process research, help ensure the success of FFF in metallic materials, making it an optimal solution for highly customized and efficient designs [84,85]. It is a worthy process, as it promptly enables the creation and simulation of thousands of designs and the production of highly customized components with complex shapes [86]. 

**Figure 5:**
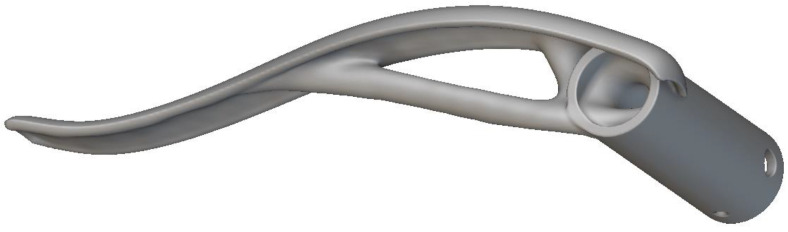
Optimized door handle optimized using DfAM techniques [77].

The DfAM approach in the FFF process (similar to other AM processes) involves designing components with the specific capabilities and constraints of the process in mind. DfAM enables the optimization of AM by leveraging its unique capabilities to create printable designs that enhance performance, functionality, and efficiency, allowing the designers to create parts optimized explicitly for AM processes, as shown in the optimized door handle in Figure 5 [77]. It differs from other design processes because it starts with an arbitrary formulation of an initial design concept and combines the algorithms’ critical structures and variables transformed by the algorithms [77,87,88]. It unlocks innovative design possibilities and overcomes traditional manufacturing limitations.

DfAM processes entail unique considerations, depending on the process to be used. In direct energy deposition (DED), a process that also uses a laser or electron beam as an energy source, designers must ensure proper cooling of the metal parts during the build process to prevent distortion and minimize the use of support structures; in PBF, thorough selection of powder material and consideration of layer thickness and build orientation is required [3,89]. DfAM to the FFF process is peculiar, takes in the limitations of the process, and considers these constraints like part of the design process, orientation, and overhangs, support structures for steep overhangs, wall thickness, part consolidation, and geometric complexity [78,90,91]. These limitations are common in AM processes and even more evident in FFF. DfAM practices are commonly implemented through specialized software, which provides insights into printability, structural integrity, and potential manufacturing issues, allowing designers to iterate and refine their models [1,49,92]. DfAM incorporates applicable requirements to address and solve the typical conflicts between design and engineering, allowing the creation of parts with well-defined functional requirements to obtain lightweight components and optimized parts by combining design and simulation tools [75,88,91]. DfAM guidelines and best practices consider the unique characteristics and limitations of AM processes, including the need for support structures, layer-by-layer build-up, and the choice of materials. 

The FFF process allows the creation of cellular structures, such as porous and lattice designs, through precise control of material deposition. Building these structures layer-by-layer, the FFF enables the infill density adjustments controlling porosity, knowing there is a trade-off between structural integrity and the level of porosity. The choice of filament material is also crucial, as it affects both the structure’s porosity and mechanical properties. Support structures may be needed to prevent collapsing during production, debinding, and sintering, and their removal can pose challenges [93,94,95]. AM lattice structures have outperformed cellular structures produced by other manufacturing methods with equivalent porosity due to the AM process’s greater geometric control and predictability [96,97,98]. By optimizing parts for AM, companies can lower production costs and turnaround times, resulting in faster product development cycles and increased competitiveness in the market. 

By adhering to DfAM guidelines, designers can create parts that perform better, cost less, and can be produced more quickly than traditional manufacturing methods [91,99]. With the continuous advancement of AM technology, the importance of DfAM is set to increase even further. DfAM is a crucial aspect of modern manufacturing. Understanding this is overwhelmingly important, as choosing the most suitable process for an intended component application is crucial in AM. Considering factors such as material properties, production volume, lead time, cost, manufacturers, and the constraints from the component requirements (like tolerances, dimensions, wall thickness, and relative density, among others) can make informed decisions and optimize their AM for the specific needs. Stating this, understanding the strengths and limitations of each AM technology is essential in selecting the most suitable one for the intended application of the component.

## 4. Challenges to Overcome from Using FFF with Metallic Materials

There are several challenges to overcome regarding FFF for metallic materials, as for other technologies for AM with metals, like PBF, DED, and binder jetting (BJT), which also present specific limitations and challenges [5,28,100,101,102].

**Figure 6:**
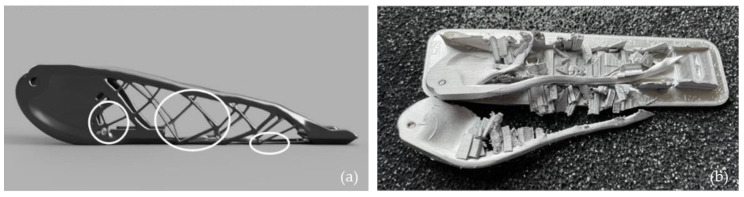
DfAM optimized component failed during the FFF process [88]. (**a**) Representative figure of an office stapler. The circles represented are the parts’ failure points. (**b**) Failed production.

Finding the correct combination between metal powders and polymer/binder systems can be challenging since it affects the response of the feedstocks, namely, melting temperatures, rheological behavior, and cooling rates. The quality of the metallic filament used in FFF can also considerably affect the final component since the distribution of the metal particles within the filament can vary, affecting the final product’s quality and mechanical properties [2,103,104]. It can be challenging to ensure that the metal particles are evenly dispersed, avoiding the particles clumping together or settling in specific areas; it can result in inconsistent production and reduced mechanical properties of the final product. FFF is susceptible to various production quality issues, including an anisotropic feature that reduces the mechanical strength of the part in specific directions (mainly in the Z-axis), warping, delamination, and poor surface finish [2,3,9,12,70,104,105]. Metal parts produced using FFF may have rough or uneven surfaces, affecting their functionality or aesthetics. It is common to find defects like pores, even between the rasterizing, and inclusions, which can also be observed on the surface [26,106]. Not all processes are suitable for producing all components, as shown in Figure 6, where an optimized office stapler failed during the FFF production phase (sintering). The circles in Figure 6a represent the failure points’ location, which is attributed to the low thickness of the lattice-like structure. The parameters should consider the process to use and aspects like the component geometry, material properties, production volume, and other factors affecting the final product’s quality and suitability.

FFF must be better suited for producing parts with complex geometry or internal structures. The layer-by-layer process can result in the formation of voids or gaps in the produced component, which can compromise its strength and integrity [2,9,70]. Heat treatments and hot isostatic pressure, which will add additional time and cost to the process, may be required to achieve the desired mechanical performance [9,103,104].

Post-processing steps like manual or automated sanding and polishing, chemical surface treatments, or surface coatings can be necessary to achieve the desired surface finish and appearance [107,108]. 

Strategies and methodologies can be employed to mitigate the post-processing in the FFF process. Starting with optimized print parameters, minimizing support structures, and implementing heat treatment for stress relief or annealing can significantly enhance the initial print quality and mechanical properties. 

While the FFF of metallic materials offers promising potential for various industries, particularly aerospace and healthcare, it presents some significant challenges, especially regarding material quality and reliability [9,109,110]. In the aerospace sector, where component integrity is paramount, FFF may need help meeting the stringent requirements for strength, durability, and material certification. Similarly, where biocompatibility and long-term reliability are essential in healthcare, FFF faces hurdles in delivering materials that meet these critical criteria [109]. The constraints linked to limited material selection, anisotropic properties, surface finish, and post-processing needs of FFF metallic materials highlight areas ripe for improvement [9,110]. Despite these challenges, there is an optimistic outlook as FFF technology continues to evolve and adapt, promising a brighter future for its application in these demanding industries. Material development and quality control advancements will undoubtedly enhance its suitability for critical applications [9,35,63,111].

## 5. The Benefits of Using FFF with Metallic Materials

FFF with metallic materials is becoming increasingly popular due to user-friendliness and cost-effective manufacturing techniques. It enables the production of parts with excellent mechanical properties comparable to conventionally manufactured parts [1,3,4,26,28,30,35,112]. In fact, FFF-produced parts can have similar tensile strength, ductility, and fatigue resistance as parts made by traditional methods, such as machining, casting, and forging [1,3,9,23]. 

The ability to produce hollow parts with reduced weight and cost is a significant advantage of metal-based FFF, which is particularly beneficial in industries like the aerospace industry, which require lightweight and durable components. 

The layer-by-layer deposition of material during the FFF process creates a characteristic microstructure that differs from conventionally manufactured parts. Adjusting sintering temperature, FFF-produced parts can have microstructures characterized by small grains, a fine distribution of strengthening phases, and small amount of defects such as voids, cracks, porosity, and inclusions [70,106,113]. Another advantage of FFF with metallic materials is its ability to produce graded materials, which vary in composition or properties over their volume, resulting in a smooth transition from one material or property to another. These graded materials can be created by changing the composition of the feedstock or adjusting the process parameters [10,114,115]. Graded materials can tailor the properties of parts to specific requirements, such as reducing weight while maintaining strength or improving wear resistance at a critical surface [114,116,117]. Ongoing research analyses the production of functionally graded materials and metal matrix composites by FFF, exploring additional applications, such as in the aerospace and biomedical industries [118,119,120].

## 6. Metallic Materials for FFF and New/Advanced Materials Utilization

Materials perform a crucial role, and FFF brings flexibility compared to beam-based and jetting processes. Advancements in materials science have led to the development of metallic filaments that can be used in FFF. These filaments are typically made by combining metal powders with a binder material, which allows the filament to be extruded through the nozzle, as previously described. Different metallic filaments can be used with FFF equipment including bronze, copper, steels, aluminum, superalloys, and titanium. More recently, new filaments combined with optimized processing conditions allowed the production of MMCs, HEAs, and FGMs. Using the unique properties of these advanced materials and taking advantage of AM makes it possible to develop high-performance components with enhanced properties tailored to specific applications. As research into these materials continues, it is expected that they will be widely adopted and used in a variety of industries around the world [8,9,121].

MMCs are composite materials that combine a metal or alloy matrix with a reinforcement material such as ceramic, metal, or fiber. These composites are known for their high strength, durability, lightweight, good thermal and electrical conductivity, and excellent wear resistance, and their utilization holds the potential to revolutionize industries like the medical, aerospace, and automotive industries [122,123]. 

FFF is a promising AM technique for producing MMCs. However, successful production of MMCs in FFF necessitates careful material selection, optimized printing parameters, uniform particle distribution, and adherence to regulatory standards. One of the most important factors is ensuring sufficient adhesion between material layers during production to maintain bonding after cooling. Other essential factors include selecting the appropriate feedstock material, controlling the temperature, and optimizing slicing parameters such as layer height and infill pattern. By considering these factors and making the appropriate adjustments, optimized FFF processes can be achieved for creating high-performing MMCs. However, additional research is needed before the widespread adoption of MMCs using FFF can occur. Developing improved processing techniques and an increased understanding of how different parameters affect material performance will facilitate increased adoption rates and result in better products at lower costs for various markets worldwide [8,121,124,125,126,127,128]. 

Unlike conventional alloys, HEAs consist of multiple principal elements, offering a wide range of compositions. HEAs possess high strength, good ductility, and improved fracture toughness. HEAs also exhibit exceptional stability at elevated temperatures, making them ideal for thermal cycling applications or high operating temperatures. Furthermore, certain HEAs outperform traditional alloys in corrosive environments, ensuring durability and longevity in challenging conditions [10,129,130,131]. HEAs are promising multi-component alloys with a unique combination of novel microstructures, and using FFF in AM will bring a new paradigm by offering unique properties and enabling new applications. The ongoing research focuses on alloy design, process optimization, feedstock development, and comprehensive characterization. FFF allows for the design and fabrication of custom HEAs tailored to the specific needs of an application, with a broad range of compositions, enabling tailored alloys for specific FFF applications. This customization can optimize properties like mechanical strength, corrosion resistance, and thermal conductivity to meet the requirements of a particular use case. 

FGMs are materials with a graded composition, microstructure, and properties, enabling them to smooth the transition between different material phases. They explore the benefits of using the FFF process by gradually changing a material’s composition, structure, or properties within a single component. Due to their unique properties, FGMs are widely used in the aerospace, automotive, and biomedical industries [132,133]. The production of FGMs using FFF involves modifying the composition of the filaments fed into the equipment, allowing the creation of custom-designed parts with varying mechanical, thermal, and electrical properties [114,133]. Furthermore, FGMs can be designed with specific characteristics, such as thermal conductivity and electrical resistivity, to address needs in different industries. For example, in the aerospace industry, FGMs can be utilized to improve the performance of jet engine components. In the biomedical sector, FGMs can be used to develop customized implants that mimic the mechanical properties of human bones. Moreover, FGMs can be used in energy harvesting devices, such as thermoelectric generators, to enhance energy conversion efficiency [134,135,136]. The production of FGMs using FFF presents several challenges, including the need for a well-controlled extrusion system to maintain a constant and precise flow of composite materials, like temperature control, feedstock homogeneity, pressure control, extruder calibration, software control, environmental conditions, and material transitions. Additionally, FFF for FGMs requires a thorough understanding of the mechanical properties and behavior of the materials involved to achieve optimal results. Nevertheless, the potential benefits of FGMs make them a promising field of research for future materials development [10,110,137,138,139].

## 7. Applications

As discussed above, FFF technology is rapidly growing in popularity and is widely adopted in various industries—aeronautical, aerospace, automotive, and medical industries, among others—and is widely used with metallic materials to create complex and lightweight components [5,16,23,140,141,142,143]. The versatility and cost-effectiveness of FFF technology make it an attractive option for a wide range of applications [23,69].

In recent years, advancements in FFF technology have made it possible to produce a range of metallic materials. FFF technology is receiving a lot of attention from the aeronautical and aerospace industries due to creating complex, lightweight components for their vehicles [78,144]. This technology can produce lightweight, high-strength components, improving fuel efficiency and reducing emissions. There are several components, such as structural components, frames, supports, sensors, and actuators for aircraft and spacecraft. FFF with metallic materials is an efficient and cost-effective way to create aircraft and spacecraft component prototypes [78]. It allows engineers to quickly design, evaluate, and modify different parts, reducing development time and costs [145,146,147]. It can also be used to create customized components that meet the specific needs of aircraft and spacecraft manufacturers. It benefits small-scale production runs or retrofitting existing aircraft or spacecraft with new components. Tooling made using FFF can be produced quickly, and using metallic materials ensures that the tools are durable and can withstand the harsh environments of the aerospace industry. Furthermore, metallic materials used in FFF can withstand high temperatures, making them suitable for creating heat-resistant components for aircraft and spacecraft [23,148]. It includes engine components, exhaust systems, and other parts exposed to high temperatures during flight. Additionally, FFF with metallic materials can be used to repair and maintain aircraft and spacecraft components, extend the life of existing components, and reduce the need for costly replacements [5,23].

FFF gained attention in the automotive industry due to its various applications. One of the most significant applications is the ability to create prototypes of new parts or components quickly. This technology enables automotive manufacturers to reduce the time and cost associated with traditional prototyping methods, making the design process more efficient. Furthermore, FFF with metallic materials can produce custom tooling for automotive manufacturing processes, which can help reduce the time and cost associated with traditional tooling methods. It can also improve fuel efficiency and performance and lower manufacturing costs [22,145,147,149]. In addition, the technology enables manufacturers to create replacement parts for older vehicles that are no longer in production, extending the life of these vehicles and reducing the need for costly repairs or replacements. Another significant benefit of FFF with metallic materials is its ability to help manufacturers reduce the weight of their vehicles while maintaining strength and durability. This can improve fuel efficiency, performance, and safety [73,150,151].

FFF technology has also emerged as a promising manufacturing process for the medical industry, particularly for prosthetics and medical devices. Using it has opened new possibilities for creating more precise and biocompatible parts and components [137,147,152]. The process enables the production of intricate parts and geometry. FFF with metallic materials offers significant advantages over conventional manufacturing methods for medical devices, such as computerized numerical control machining and casting. The technology is more cost-effective, has shorter lead times, and offers greater design flexibility. It is significant to produce custom implants, which must be tailored to the patient’s anatomy [137,147,152]. One of the most important applications of FFF with metallic materials in the medical industry is the production of implantable medical devices, such as dental, cranial, and spinal implants. These implants must be biocompatible, durable, and have precise geometries that match the patient’s anatomy. FFF technology is an effective method for producing such implants with high precision and biocompatibility. The use of metallic materials in FFF also enables the production of implants with high strength and corrosion resistance, which are crucial for long-term implant stability [44,153,154,155]. FFF with metallic materials has also been used to create surgical instruments, such as scalpels, forceps, and tweezers. The technology enables the production of surgical instruments with complex geometries and customized designs. The devices produced have also been found to have good mechanical properties, such as high strength and wear resistance, similar to conventional materials. Summarizing, FFF with metallic materials has immense potential in the medical industry, particularly for producing prosthetics and medical devices, and can help improve patient care quality and advance medical innovation by enabling the production of precise and biocompatible parts and components. The technology offers several advantages over conventional manufacturing methods, including cost-effectiveness, faster turnaround times, and greater design flexibility [137,147,156]. 

The tool and mold-making industry has found numerous applications for FFF technology, including prototyping, creating jigs and fixtures, low-volume production, and custom tooling [18,157]. FFF technology enables designers and manufacturers to make quick and inexpensive prototypes of tools and molds before investing in expensive tooling. It can also be used to create custom jigs and fixtures that hold workpieces in place during manufacturing processes. These jigs and fixtures can be designed and quickly produced, which reduces lead time and costs [63,104,147,158]. FFF technology is helpful for low-volume production of tooling components, such as inserts, cores, and cavities, particularly for small businesses that cannot afford expensive tooling or require frequent design changes. Custom tooling is another application of FFF technology, where the molds can be designed to create unique shapes or textures that are difficult to achieve with traditional molding techniques. FFF technology has revolutionized the tool and mold-making industry, offering rapid prototyping, low-volume production, tailored tools, and molds, significantly reducing lead times and costs. The ability to easily create customized and complex designs is instrumental in tooling maintenance, allowing the creation of replacement components. In today’s manufacturing landscape, FFF has become an indispensable tool for streamlining processes, reducing costs, and maintaining competitiveness. The FFF technology has become an increasingly popular option for manufacturers looking to improve their productivity and reduce costs [18,21,44,70,159].

Additionally, several other industries employ FFF with metallic materials as a successful AM technique, including marine engineering, architecture and construction, jewelry and watchmaking, art and sculpture, electronics manufacturing, and sports equipment manufacturing. As stated before, the versatility and cost-effectiveness allowed by the FFF technology make it an appealing alternative for various applications [27,159,160].

## 8. Conclusions

FFF of metallic materials has shown enormous potential to revolutionize how metallic parts are produced. Its versatility and cost-effectiveness offer promising technology for various industries and applications. Metal-based AM can help create more efficient, cost-effective, and sustainable products, enabling greater customization, faster prototyping, and lighter-weight components. AM technology can transform industries from aeronautical to aerospace, automotive, medical, and others. Ongoing research and development are expected to lead to further improvements in the technology and expand its range of applications. FFF of metallic materials is an exciting area of research with the potential to transform industries by enabling the production of complex, custom parts with reduced lead times and costs. Even though it has advantages, several challenges still need to be addressed. One of the most significant challenges is the limited range of metallic materials available as filaments. The materials available for FFF of metallic materials are limited compared to other metal-based AM processes. This limits the application of FFF of metallic materials to specific parts and industries. Furthermore, the size of the produced parts is limited, and the post-processing requirements can be significant. Additionally, the manufacturing orientation and support structures can affect the properties of the printed metallic parts, requiring careful consideration.

Researchers are exploring new techniques and materials, including hybrid systems that combine FFF with other AM technologies, to address the challenges of limited material range, size limitations, and post-processing requirements. MMCs and FGMs offer a promising area of research that can enable the creation of custom parts with varying mechanical properties. FFF of metallic materials is a technology to watch in the coming years with the potential to create more efficient, cost-effective, and sustainable products. The continued research and development of FFF of metallic materials will enable the creation of more complex and custom parts, further revolutionizing manufacturing across various industries. With MMCs and FGMs, this technology can create custom-made parts with varying mechanical properties, such as stiffness, strength, and thermal conductivity, which can benefit various applications. 

The widespread adoption of metal-based FFF could significantly impact the manufacturing industry. Metal-based FFF has the potential to democratize manufacturing by enabling small businesses and individuals to produce custom parts on demand. This could significantly impact the supply chain, reducing the need for large-scale manufacturing facilities and enabling greater customization of products. Additionally, metal-based FFF can potentially reduce the carbon footprint of the manufacturing industry by reducing waste and enabling the creation of lighter-weight products.

In summary, the FFF of metallic materials is a promising technology with several advantages, including its ease of use, affordability, and versatility. This technology has the potential to revolutionize manufacturing across a range of industries. However, several challenges still need to be addressed, including the limited scope of metallic materials available as filaments, the limited size of the printed parts, and the post-processing requirements. Continued research and development are needed to overcome the remaining challenges and enable the widespread use of FFF to produce metallic parts.

## 9. Prospects for the Metal FFF Future

One of the most significant prospects for metal-based FFF is developing new materials. Currently, the range of commercial metallic materials available as filaments for FFF is limited, which restricts the range of applications that can be achieved. However, ongoing research and development are expected to lead to new materials suitable for FFF, expanding the range of applications that can be achieved. In the materials, it is also critical to investigate and find new binders, more adapted to FFF and the processes of removal of binder and sintering to enable better final components. New binders are the next step to revolutionize FFF for metallic materials, playing a pivotal role in the process by offering the potential for superior final component quality. The main properties required are a strong adhesion, ensuring precise extrusion to avoid parts deformation during the FFF process, and easy debinding. Therefore, they are critical for ensuring a more sustainable debinding process with a smaller environmental impact, promoting waste reduction.

To achieve the desired properties, the parts must also be subjected to post-processing, such as heat treatment or machining. This can be time-consuming and expensive, reducing the cost-effectiveness of the technology. Continued research and development are needed to develop new post-processing techniques that are more cost-effective and time-efficient. Another significant challenge is the manufacturing orientation and support structures required for metal-based FFF. The orientation of the part and the support structures used can affect the properties of the printed part, requiring careful consideration during the design process. Continued research and development are needed to develop new software and hardware to optimize the printing process and reduce the need for support structures. 

The improvement of the manufacturing process itself is required. One of the most significant challenges is the limited size of the produced parts. Continued research and development are expected to lead to equipment development with larger build volume, facilitating the creation of larger and more complex metal parts in a single build. This promotes versatility and time saving when producing big parts, eliminating smaller components’ assembly operations and reducing labor and potential points of failure. 

Additionally, improvements in the development of new software and hardware are expected to improve the precision and accuracy of the printing process, enabling the creation of more complex parts with greater accuracy. Also, improving the debinding and sintering process is necessary to allow the production of better components more quickly and feasibly. Additionally, research is being conducted on hybrid systems that combine FFF with other AM technologies, such as PBF or DED processes, to create new materials with enhanced properties, which offer substantial advantages in terms of mechanical properties and material versatility. These systems offer advantages in terms of tighter tolerances, improved surface finishes, and a wider material range for complex geometries. They hold potential for aerospace, automotive, and healthcare applications, providing customizability, cost-efficiency, and rapid prototyping capabilities.

## Data Availability

Not applicable.

## Nomenclature

AM	Additive manufacturing
BJT	Binder jetting
CAD	Computer-aided design
DED	Direct energy deposition
DfAM	Design for additive manufacturing
FFF	Fused filament fabrication
FGM	Functionally graded materials
HEA	High entropy alloys
MEX	Material extrusion
MMC	Metal matrix composites
PBF	Powder bed fusion
TO	Topology optimization

## References

[1] DebRoy T., Wei H.L., Zuback J.S., Mukherjee T., Elmer J.W., Milewski J.O., Beese A.M., Wilson-Heid A., De A., Zhang W. (2018). Additive manufacturing of metallic components—Process, structure and properties. Prog. Mater. Sci..

[2] Tuncer N., Bose A. (2020). Solid-State Metal Additive Manufacturing: A Review. JOM.

[3] Costa J., Sequeiros E., Vieira M.T., Vieira M. (2021). Additive Manufacturing: Material Extrusion of Metallic Parts. U. Porto J. Eng..

[4] Herderick E. Additive Manufacturing of Metals: A Review. Proceedings of the MS&T 2011: Proceedings from the Materials Science & Technology Conference.

[5] Gibson I., Rosen D.W., Stucker B. (2010). Additive Manufacturing Technologies: Rapid Prototyping to Direct Digital Manufacturing.

[6] Gonzalez-Gutierrez J., Cano S., Schuschnigg S., Kukla C., Sapkota J., Holzer C. (2018). Additive Manufacturing of Metallic and Ceramic Components by the Material Extrusion of Highly-Filled Polymers: A Review and Future Perspectives. Materials.

[7] Dey A., Roan Eagle I.N., Yodo N. (2021). A Review on Filament Materials for Fused Filament Fabrication. J. Manuf. Mater. Proc..

[8] Behera M.P., Dougherty T., Singamneni S. (2019). Conventional and Additive Manufacturing with Metal Matrix Composites: A Perspective. Digit. Manuf. Transform. Ind. Towards Sustain. Growth.

[9] Tofail S.A.M., Koumoulos E.P., Bandyopadhyay A., Bose S., O’Donoghue L., Charitidis C. (2018). Additive manufacturing: Scientific and technological challenges, market uptake and opportunities. Mater. Today.

[10] Zhang C., Chen F., Huang Z., Jia M., Chen G., Ye Y., Lin Y., Liu W., Chen B., Shen Q. (2019). Additive manufacturing of functionally graded materials: A review. Mater. Sci. Eng. A.

[11] Zhang Y.Z., Bai S.Y., Riede M., Garratt E., Roch A. (2020). A comprehensive study on fused filament fabrication of Ti-6Al-4V structures. Addit. Manuf..

[12] Kok Y., Tan X.P., Wang P., Nai M.L.S., Loh N.H., Liu E., Tor S.B. (2018). Anisotropy and heterogeneity of microstructure and mechanical properties in metal additive manufacturing: A critical review. Mater. Des..

[13] Chern A.H., Nandwana P., McDaniels R., Dehoff R.R., Liaw P.K., Tryon R., Duty C.E. (2020). Build orientation, surface roughness, and scan path influence on the microstructure, mechanical properties, and flexural fatigue behavior of Ti–6Al–4V fabricated by electron beam melting. Mater. Sci. Eng. A.

[14] Chen L.Y., Huang J.C., Lin C.H., Pan C.T., Chen S.Y., Yang T.L., Lin D.Y., Lin H.K., Jang J.S.C. (2017). Anisotropic response of Ti-6Al-4V alloy fabricated by 3D printing selective laser melting. Mater. Sci. Eng. A.

[15] Leach R.K., Bourell D., Carmignato S., Donmez A., Senin N., Dewulf W. (2019). Geometrical metrology for metal additive manufacturing. CIRP Ann.—Manuf. Technol..

[16] Atzeni E., Salmi A. (2012). Economics of additive manufacturing for end-usable metal parts. Int. J. Adv. Manuf. Technol..

[17] Atzeni E., Salmi A. (2015). Study on unsupported overhangs of AlSi10Mg parts processed by Direct Metal Laser Sintering (DMLS). J. Manuf. Process..

[18] Boparai K.S., Singh R., Singh H. (2016). Development of rapid tooling using fused deposition modeling: A review. Rapid Prototyp. J.

[19] (2019). Standard Terminology for Additive Manufacturing—Coordinate Systems and Test Methodologies.

[20] Shaik Y.P., Schuster J., Shaik A. (2021). A Scientific Review on Various Pellet Extruders Used In 3D Printing FDM Processes. Open Access Libr. J..

[21] Kumar N., Jain P.K., Tandon P., Pandey P.M. (2018). Extrusion-based additive manufacturing process for producing flexible parts. J. Braz. Soc. Mech. Sci. Eng..

[22] Boyle B.M., Xiong P.T., Mensch T.E., Werder T.J., Miyake G.M. (2019). 3D printing using powder melt extrusion. Addit. Manuf..

[23] Gibson M.A., Mykulowycz N.M., Shim J., Fontana R., Schmitt P., Roberts A., Ketkaew J., Shao L., Chen W., Bordeenithikasem P. (2018). 3D printing metals like thermoplastics: Fused filament fabrication of metallic glasses. Mater. Today.

[24] Matsuzaki R., Kanatani T., Todoroki A. (2019). Multi-material additive manufacturing of polymers and metals using fused filament fabrication and electroforming. Addit. Manuf..

[25] Annoni M., Giberti H., Strano M. (2016). Feasibility Study of an Extrusion-based Direct Metal Additive Manufacturing Technique. Procedia Manuf..

[26] Thompson Y., Gonzalez-Gutierrez J., Kukla C., Felfer P. (2019). Fused filament fabrication, debinding and sintering as a low cost additive manufacturing method of 316L stainless steel. Addit. Manuf..

[27] Gonzalez-Gutierez J., Godec D., Guran R., Spoerk M., Kukla C., Holzer C. (2018). 3d Printing Conditions Determination for Feedstock Used in Fused Filament Fabrication (Fff) of 17-4ph Stainless Steel Parts. Metalurgija.

[28] Frazier W.E. (2014). Metal Additive Manufacturing: A Review. J. Mater. Eng. Perform..

[29] Thompson S.M., Bian L., Shamsaei N., Yadollahi A. (2015). An overview of Direct Laser Deposition for additive manufacturing; Part I: Transport phenomena, modeling and diagnostics. Addit. Manuf..

[30] Angelopoulos P.M., Samouhos M., Taxiarchou M. (2021). Functional fillers in composite filaments for fused filament fabrication; a review. Mater. Today Proc..

[31] Singh P., Balla V.K., Tofangchi A., Atre S.V., Kate K.H. (2020). Printability studies of Ti-6Al-4V by metal fused filament fabrication (MF3). Int. J. Refract. Met. Hard Mater..

[32] Schatt W., Wieters K.-P. (1997). Powder Metallurgy—Processing and Materials.

[33] Cano S., Gonzalez-Gutierrez J., Sapkota J., Spoerk M., Arbeiter F., Schuschnigg S., Holzer C., Kukla C. (2019). Additive manufacturing of zirconia parts by fused filament fabrication and solvent debinding: Selection of binder formulation. Addit. Manuf..

[34] Vock S., Klöden B., Kirchner A., Weißgärber T., Kieback B. (2019). Powders for powder bed fusion: A review. Prog. Addit. Manuf..

[35] Wagner M.A., Hadian A., Sebastian T., Clemens F., Schweizer T., Rodriguez-Arbaizar M., Carreño-Morelli E., Spolenak R. (2022). Fused filament fabrication of stainless steel structures—From binder development to sintered properties. Addit. Manuf..

[36] Diegel O., Nordin A., Motte D. (2019). Additive Manufacturing Technologies.

[37] Gibson I., Rosen D., Stucker B., Khorasani M. (2021). Introduction and Basic Principles. Additive Manufacturing Technologies.

[38] Sequeiros E.W., Emadinia O., Vieira M.T., Vieira M.F. (2020). Development of Metal Powder Hot Embossing: A New Method for Micromanufacturing. Metals.

[39] Singh P., Shaikh Q., Balla V.K., Atre S.V., Kate K.H. (2019). Estimating Powder-Polymer Material Properties Used in Design for Metal Fused Filament Fabrication (DfMF3). JOM.

[40] Emadinia O., Vieira M.T., Vieira M.F. (2021). Development and characterization of AISI 316L micro parts produced by metal powder hot embossing. Int. J. Adv. Manuf. Technol..

[41] Sequeiros E.W., Ferreira T.J., Neto V.C., Vieira M.T., Vieira M.F. (2015). Microstructural Characterization of Metallic Parts Produced by Hot Embossing. Microsc. Microanal..

[42] Singh S., Prakash C., Antil P., Singh R., Krolczyk G., Pruncu C.I. (2019). Dimensionless Analysis for Investigating the Quality Characteristics of Aluminium Matrix Composites Prepared through Fused Deposition Modelling Assisted Investment Casting. Materials.

[43] Royer A., Barriere T., Gelin J.C. (2016). Development and Characterization of a Metal Injection Molding Bio Sourced Inconel 718 Feedstock Based on Polyhydroxyalkanoates. Metals.

[44] Singh S., Ramakrishna S., Singh R. (2017). Material issues in additive manufacturing: A review. J. Manuf. Process..

[45] Volpato N., Kretschek D., Foggiatto J.A., Cruz C.M.G.D. (2015). Experimental analysis of an extrusion system for additive manufacturing based on polymer pellets. Int. J. Adv. Manuf. Technol..

[46] Zhou Z., Salaoru I., Morris P., Gibbons G.J. (2018). Additive manufacturing of heat-sensitive polymer melt using a pellet-fed material extrusion. Addit. Manuf..

[47] Whyman S., Arif K.M., Potgieter J. (2018). Design and development of an extrusion system for 3D printing biopolymer pellets. Int. J. Adv. Manuf. Technol..

[48] Xu C., Quinn B., Lebel L.L., Therriault D., L’Espérance G. (2019). Multi-Material Direct Ink Writing (DIW) for Complex 3D Metallic Structures with Removable Supports. ACS Appl. Mater. Interfaces.

[49] Ang X., Tey J.Y., Yeo W.H., Shak K.P.Y. (2023). A review on metallic and ceramic material extrusion method: Materials, rheology, and printing parameters. J. Manuf. Process..

[50] Pandya K.S., Shindalkar S.S., Kandasubramanian B. (2023). Breakthrough to the pragmatic evolution of direct ink writing: Progression, challenges, and future. Prog. Addit. Manuf..

[51] Bonada J., Xuriguera E., Calvo L., Poudelet L., Cardona R., Padilla J.A., Niubó M., Fenollosa F. (2019). Analysis of printing parameters for metal additive manufactured parts through Direct Ink Writing process. Procedia Manuf..

[52] Saadi M.A.S.R., Maguire A., Pottackal N.T., Thakur M.S.H., Ikram M.M., Hart A.J., Ajayan P.M., Rahman M.M. (2022). Direct Ink Writing: A 3D Printing Technology for Diverse Materials. Adv. Mater..

[53] Elkaseer A., Chen K.J., Janhsen J.C., Refle O., Hagenmeyer V., Scholz S.G. (2022). Material jetting for advanced applications: A state-of-the-art review, gaps and future directions. Addit. Manuf..

[54] Balani S.B., Ghaffar S.H., Chougan M., Pei E., Şahin E. (2021). Processes and materials used for direct writing technologies: A review. Results Eng..

[55] Armstrong M., Mehrabi H., Naveed N. (2022). An overview of modern metal additive manufacturing technology. J. Manuf. Process..

[56] Mostafaei A., Steven E.L., Ference J.J., Schmid D.E., Chmielus M. (2018). Binder jetting of a complex-shaped metal partial denture framework. Addit. Manuf..

[57] Mostafaei A., Elliott A.M., Barnes J.E., Li F., Tan W., Cramer C.L., Nandwana P., Chmielus M. (2020). Binder jet 3D printing—Process parameters, materials, properties, modeling, and challenges. Prog. Mater. Sci..

[58] Mostafaei A., Neelapu S.H.V.R., Kisailus C., Nath L.M., Jacobs T.D.B., Chmielus M. (2018). Characterizing surface finish and fatigue behavior in binder-jet 3D-printed nickel-based superalloy 625. Addit. Manuf..

[59] Al C.M., Yaman U. (2017). Improving the strength of additively manufactured objects via modified interior structure. AIP Conf. Proc..

[60] Gao W., Zhang Y.B., Ramanujan D., Ramani K., Chen Y., Williams C.B., Wang C.C.L., Shin Y.C., Zhang S., Zavattieri P.D. (2015). The status, challenges, and future of additive manufacturing in engineering. Comput. Aided. Des..

[61] Rane K., Strano M. (2019). A comprehensive review of extrusion-based additive manufacturing processes for rapid production of metallic and ceramic parts. Adv. Manuf..

[62] Thompson M.K., Moroni G., Vaneker T., Fadel G., Campbell R.I., Gibson I., Bernard A., Schulz J., Graf P., Ahuja B. (2016). Design for Additive Manufacturing: Trends, opportunities, considerations, and constraints. CIRP. Ann.—Manuf. Technol..

[63] Turner B.N., Strong R., Gold S.A. (2014). A review of melt extrusion additive manufacturing processes: I. Process design and modeling. Rapid Prototyp. J..

[64] Greeff G.P., Schilling M. (2018). Single print optimisation of fused filament fabrication parameters. Int. J. Adv. Manuf. Technol..

[65] Hertle S., Kleffel T., Worz A., Drummer D. (2020). Production of polymer-metal hybrids using extrusion-based additive manufacturing and electrochemically treated aluminum. Addit. Manuf..

[66] Kumar L.J., Pandey P.M., Wimpenny D.I. (2019). 3D Printing and Additive Manufacturing Technologies.

[67] Patel A., Taufik M. (2022). Extrusion-Based Technology in Additive Manufacturing: A Comprehensive Review. Arab. J. Sci. Eng..

[68] Hui W., Shao C., Zhang Y., Zhao X., Weng Y. (2017). Microstructure and mechanical properties of medium Mn steel containing 3%Al processed by warm rolling. Mater. Sci. Eng. A.

[69] Nath P., Olson J.D., Mahadevan S., Lee Y.-T.T. (2020). Optimization of fused filament fabrication process parameters under uncertainty to maximize part geometry accuracy. Addit. Manuf..

[70] Yadav A., Rohru P., Babbar A., Kumar R., Ranjan N., Chohan J.S., Kumar R., Gupta M. (2022). Fused filament fabrication: A state-of-the-art review of the technology, materials, properties and defects. Int. J. Interact. Des. Manuf. (IJIDeM).

[71] Notzel D., Hanemann T. (2020). New Feedstock System for Fused Filament Fabrication of Sintered Alumina Parts. Materials.

[72] Mulholland T., Goris S., Boxleitner J., Osswald T.A., Rudolph N. (2018). Fiber Orientation Effects in Fused Filament Fabrication of Air-Cooled Heat Exchangers. JOM J. Miner. Met. Mater. Soc. (TMS).

[73] Zanjanijam A.R., Major I., Lyons J.G., Lafont U., Devine D.M. (2020). Fused Filament Fabrication of PEEK: A Review of Process-Structure-Property Relationships. Polymers.

[74] Li S., Wei H., Yuan S., Zhu J., Li J., Zhang W. (2023). Collaborative optimization design of process parameter and structural topology for laser additive manufacturing. Chin. J. Aeronaut..

[75] Gebisa A.W., Lemu H.G. (2017). Design for manufacturing to design for Additive Manufacturing: Analysis of implications for design optimality and product sustainability. Procedia Manuf..

[76] Sossou G., Demoly F., Montavon G., Gomes S. (2017). An additive manufacturing oriented design approach to mechanical assemblies. J. Comput. Des. Eng..

[77] Mata M., Pinto M., Costa J. (2023). Topological Optimization of a Metal Extruded Doorhandle using nTopology. U. Porto J. Eng..

[78] Schmelzle J., Kline E.V., Dickman C.J., Reutzel E.W., Jones G., Simpson T.W. (2015). (Re)Designing for Part Consolidation: Understanding the Challenges of Metal Additive Manufacturing. J. Mech. Des..

[79] Leary M., Downing D., Lozanovski B., Harris J., Yadroitsev I., Yadroitsava I., du Plessis A., MacDonald E. (2021). 5—Design principles. Fundamentals of Laser Powder Bed Fusion of Metals.

[80] Dara A., Johnney Mertens A., Raju Bahubalendruni M.V.A. (2022). Characterization of penetrate and interpenetrate tessellated cellular lattice structures for energy absorption. Proc. Inst. Mech. Eng. Part L J. Mater. Des. Appl..

[81] Chouhan G., Gunji B.M., Bidare P., Ramakrishna D., Kumar R. (2023). Experimental and numerical investigation of 3D printed bio-inspired lattice structures for mechanical behaviour under Quasi static loading conditions. Mater. Today Commun..

[82] Ramakrishna D., Bala Murali G. (2022). Bio-inspired 3D-printed lattice structures for energy absorption applications: A review. Proc. Inst. Mech. Eng. Part L J. Mater. Des. Appl..

[83] Mazur M., Leary M., McMillan M., Sun S., Shidid D., Brandt M. (2017). Mechanical properties of Ti6Al4V and AlSi12Mg lattice structures manufactured by Selective Laser Melting (SLM). Laser Additive Manufacturing.

[84] Nyamekye P., Unt A., Salminen A., Piili H. (2020). Integration of Simulation Driven DfAM and LCC Analysis for Decision Making in L-PBF. Metals.

[85] McMillan M., Leary M., Brandt M. (2017). Computationally efficient finite difference method for metal additive manufacturing: A reduced-order DFAM tool applied to SLM. Mater. Des..

[86] Vuillemot R., Huron S. Glitches as a generative design process. Proceedings of the 2017 IEEE VIS Arts Program (VISAP).

[87] Cui J., Tang M.X. (2017). Towards generative systems for supporting product design. Int. J. Des. Eng..

[88] Oliveira C., Maia M., Costa J. (2023). Production of an Office Stapler by Material Extrusion Process, using DfAM as Optimization Strategy. U. Porto J. Eng..

[89] Wang X., Xu S., Zhou S., Xu W., Leary M., Choong P., Qian M., Brandt M., Xie Y.M. (2016). Topological design and additive manufacturing of porous metals for bone scaffolds and orthopaedic implants: A review. Biomaterials.

[90] Mass Y., Amir O. (2017). Topology optimization for additive manufacturing: Accounting for overhang limitations using a virtual skeleton. Addit. Manuf..

[91] Yang S., Zhao Y.F. (2015). Additive manufacturing-enabled design theory and methodology: A critical review. Int. J. Adv. Manuf. Technol..

[92] Langelaar M. (2017). An additive manufacturing filter for topology optimization of print-ready designs. Struct. Multidiscip. Optim..

[93] Dara A., Bahubalendruni M.V.A.R., Johnney Mertens A., Balamurali G. (2022). Numerical and experimental investigations of novel nature inspired open lattice cellular structures for enhanced stiffness and specific energy absorption. Mater. Today Commun..

[94] Samson S., Tran P., Marzocca P. (2023). Design and modelling of porous gyroid heatsinks: Influences of cell size, porosity and material variation. Appl. Therm. Eng..

[95] Riza S.H., Masood S.H., Wen C.E., Ruan D., Xu S.Q. (2014). Dynamic behaviour of high strength steel parts developed through laser assisted direct metal deposition. Mater. Des..

[96] Maconachie T., Leary M., Lozanovski B., Zhang X., Qian M., Faruque O., Brandt M. (2019). SLM lattice structures: Properties, performance, applications and challenges. Mater. Des..

[97] Cheng L., Zhang P., Biyikli E., Bai J., Pilz S., To A.C. Integration of topology optimization with efficient design of additive manufactured cellular structures. Proceedings of the Solid Freeform Fabrication (SFF) Conference.

[98] Park S.I., Rosen D.W., Choi S.K., Duty C.E. Effective Mechanical Properties of Lattice Material Fabricated by Material Extrusion Additive Manufacturing. Proceedings of the ASME International Design Engineering Technical Conferences and Computers and Information in Engineering Conference.

[99] Yang L., Hsu K., Baughman B., Godfrey D., Medina F., Menon M., Wiener S. (2017). Additive Manufacturing of Metals: The Technology, Materials, Design and Production.

[100] Huang Y., Leu M.C., Mazumder J., Donmez A. (2015). Additive Manufacturing: Current State, Future Potential, Gaps and Needs, and Recommendations. J. Manuf. Sci. E-T ASME.

[101] Cooke S., Ahmadi K., Willerth S., Herring R. (2020). Metal additive manufacturing: Technology, metallurgy and modelling. J. Manuf. Process..

[102] Ciurana J. (2013). New Opportunities and Challenges for Additive Manufacturing to Produce Biomedical Devices. IFAC Proc. Vol..

[103] Haghdadi N., Laleh M., Moyle M., Primig S. (2021). Additive manufacturing of steels: A review of achievements and challenges. J. Mater. Sci..

[104] Turner B.N., Gold S.A. (2015). A review of melt extrusion additive manufacturing processes: II. Materials, dimensional accuracy, and surface roughness. Rapid. Prototyp. J..

[105] Wang D., Han H., Sa B., Li K., Yan J., Zhang J., Liu J., He Z., Wang N., Yan M. (2022). A review and a statistical analysis of porosity in metals additively manufactured by laser powder bed fusion. Opto-Electron. Adv..

[106] Tosto C., Tirillò J., Sarasini F., Cicala G. (2021). Hybrid Metal/Polymer Filaments for Fused Filament Fabrication (FFF) to Print Metal Parts. Appl. Sci..

[107] Ge J., Pillay S., Ning H. (2023). Post-Process Treatments for Additive-Manufactured Metallic Structures: A Comprehensive Review. J. Mater. Eng. Perform..

[108] Mu J., Sun T., Leung C.L.A., Oliveira J.P., Wu Y., Wang H., Wang H. (2023). Application of electrochemical polishing in surface treatment of additively manufactured structures: A review. Prog. Mater. Sci..

[109] Javaid M., Haleem A., Singh R.P., Suman R. (2022). 3D printing applications for healthcare research and development. Glob. Health J..

[110] Nazir A., Gokcekaya O., Md Masum Billah K., Ertugrul O., Jiang J., Sun J., Hussain S. (2023). Multi-material additive manufacturing: A systematic review of design, properties, applications, challenges, and 3D printing of materials and cellular metamaterials. Mater. Des..

[111] Chua C.K., Wong C.H., Yeong W.Y., Chua C.K., Wong C.H., Yeong W.Y. (2017). Chapter Five—Material Characterization for Additive Manufacturing. Standards, Quality Control, and Measurement Sciences in 3D Printing and Additive Manufacturing.

[112] Cuan-Urquizo E., Barocio E., Tejada-Ortigoza V., Pipes R., Rodriguez C., Roman-Flores A. (2019). Characterization of the Mechanical Properties of FFF Structures and Materials: A Review on the Experimental, Computational and Theoretical Approaches. Materials.

[113] Coogan T.J., Kazmer D.O. (2020). Prediction of interlayer strength in material extrusion additive manufacturing. Addit. Manuf..

[114] Sam M., Jojith R., Radhika N. (2021). Progression in manufacturing of functionally graded materials and impact of thermal treatment—A critical review. J. Manuf. Process..

[115] Reichardt A., Shapiro A.A., Otis R., Dillon R.P., Borgonia J.P., McEnerney B.W., Hosemann P., Beese A.M. (2021). Advances in additive manufacturing of metal-based functionally graded materials. Int. Mater. Rev..

[116] Ostolaza M., Arrizubieta J.I., Lamikiz A., Plaza S., Ortega N. (2023). Latest Developments to Manufacture Metal Matrix Composites and Functionally Graded Materials through AM: A State-of-the-Art Review. Materials.

[117] Loh G.H., Pei E.J., Harrison D., Monzon M.D. (2018). An overview of functionally graded additive manufacturing. Addit. Manuf..

[118] Traxel K.D., Bandyopadhyay A. (2018). Reactive-deposition-based additive manufacturing of Ti-Zr-BN composites. Addit. Manuf..

[119] Li Y., Feng Z., Hao L., Huang L., Xin C., Wang Y., Bilotti E., Essa K., Zhang H., Li Z. (2020). A Review on Functionally Graded Materials and Structures via Additive Manufacturing: From Multi-Scale Design to Versatile Functional Properties. Adv. Mater. Technol..

[120] Bobbio L.D., Bocklund B., Otis R., Borgonia J.P., Dillon R.P., Shapiro A.A., McEnerney B., Liu Z.-K., Beese A.M. (2018). Characterization of a functionally graded material of Ti-6Al-4V to 304L stainless steel with an intermediate V section. J. Alloys Compd..

[121] Togwe T., Gokce A., Chen Y.Y., German R.M., Atre S. (2020). Metal matrix composites for fabricating tooling. Int. J. Refract. Met. Hard Mater..

[122] Rahman M., Islam K.S., Dip T.M., Chowdhury M.F.M., Debnath S.R., Hasan S.M.M., Sakib M.S., Saha T., Padhye R., Houshyar S. (2023). A review on nanomaterial-based additive manufacturing: Dynamics in properties, prospects, and challenges. Prog. Addit. Manuf..

[123] Zhang W., Xu J. (2022). Advanced lightweight materials for Automobiles: A review. Mater. Des..

[124] Sheydaeian E., Toyserkani E. (2018). A new approach for fabrication of titanium-titanium boride periodic composite via additive manufacturing and pressure-less sintering. Compos. Part B—Eng..

[125] Mostafaei A., Heidarzadeh A., Brabazon D. (2020). Additive Manufacturing of Metal Matrix Composites. Reference Module in Materials Science and Materials Engineering.

[126] Mostafaei A., Heidarzadeh A., Brabazon D. (2021). Production of Metal Matrix Composites Via Additive Manufacturing. Reference Module in Materials Science and Materials Engineering.

[127] Sharma D.K., Mahant D., Upadhyay G. (2020). Manufacturing of metal matrix composites: A state of review. Mater. Today Proc..

[128] Sharma A.K., Bhandari R., Aherwar A., Rimašauskienė R. (2020). Matrix materials used in composites: A comprehensive study. Mater. Today Proc..

[129] Ron T., Shirizly A., Aghion E. (2023). Additive Manufacturing Technologies of High Entropy Alloys (HEA): Review and Prospects. Materials.

[130] Ye Y.F., Wang Q., Lu J., Liu C.T., Yang Y. (2016). High-entropy alloy: Challenges and prospects. Mater. Today.

[131] Sheikh S., Gan L., Tsao T.-K., Murakami H., Shafeie S., Guo S. (2018). Aluminizing for enhanced oxidation resistance of ductile refractory high-entropy alloys. Intermetallics.

[132] Ghanavati R., Naffakh-Moosavy H. (2021). Additive Manufacturing of Functionally Graded Metallic Materials: A Review of Experimental and Numerical Studies. J. Mater. Res. Technol..

[133] Boggarapu V., Gujjala R., Ojha S., Acharya S., Venkateswara Babu P., Chowdary S., kumar Gara D. (2021). State of the art in functionally graded materials. Compos. Struct..

[134] Ren L., Wang Z., Ren L., Han Z., Liu Q., Song Z. (2022). Graded biological materials and additive manufacturing technologies for producing bioinspired graded materials: An overview. Compos. Part B Eng..

[135] El-Galy I.M., Saleh B.I., Ahmed M.H. (2019). Functionally graded materials classifications and development trends from industrial point of view. SN Appl. Sci..

[136] Naebe M., Shirvanimoghaddam K. (2016). Functionally graded materials: A review of fabrication and properties. Appl. Mater. Today.

[137] Hasanov S., Gupta A., Nasirov A., Fidan I. (2020). Mechanical characterization of functionally graded materials produced by the fused filament fabrication process. J. Manuf. Process..

[138] Hasanov S., Gupta A., Alifui-Segbaya F., Fidan I. (2021). Hierarchical homogenization and experimental evaluation of functionally graded materials manufactured by the fused filament fabrication process. Compos. Struct..

[139] Chueh Y.H., Wei C., Zhang X.J., Li L. (2020). Integrated laser-based powder bed fusion and fused filament fabrication for three-dimensional printing of hybrid metal/polymer objects. Addit. Manuf..

[140] Careri F., Khan R.H.U., Todd C., Attallah M.M. (2023). Additive manufacturing of heat exchangers in aerospace applications: A review. Appl. Therm. Eng..

[141] Bandyopadhyay A., Heer B. (2018). Additive manufacturing of multi-material structures. Mater. Sci. Eng. R Rep..

[142] Siddique S., Imran M., Rauer M., Kaloudis M., Wycisk E., Emmelmann C., Walther F. (2015). Computed tomography for characterization of fatigue performance of selective laser melted parts. Mater. Des..

[143] Gu D.D., Meiners W., Wissenbach K., Poprawe R. (2012). Laser additive manufacturing of metallic components: Materials, processes and mechanisms. Int. Mater. Rev..

[144] Petrovic V., Ninerola R. (2015). Powder recyclability in electron beam melting for aeronautical use. Aircr. Eng. Aerosp. Technol..

[145] Harris M., Potgieter J., Archer R., Arif K.M. (2019). Effect of Material and Process Specific Factors on the Strength of Printed Parts in Fused Filament Fabrication: A Review of Recent Developments. Materials.

[146] Waalkes L., Langerich J., Holbe F., Emmelmann C. (2020). Feasibility study on piston-based feedstock fabrication with Ti-6Al-4V metal injection molding feedstock. Addit. Manuf..

[147] Warrier N., Kate K.H. (2018). Fused filament fabrication 3D printing with low-melt alloys. Prog. Addit. Manuf..

[148] Gorelik M. (2017). Additive manufacturing in the context of structural integrity. Int. J. Fatigue.

[149] Hinduja S., Fan K.-C., Hon K.K.B. Digital Additive Manufacturing: From Rapid Prototyping to Rapid Manufacturing. Proceedings of the 35th International MATADOR Conference: Formerly The International Machine Tool Design and Research Conference.

[150] Yang G.Q., Mo J.K., Kang Z.Y., Dohrmann Y., List F.A., Green J.B., Babu S.S., Zhang F.Y. (2018). Fully printed and integrated electrolyzer cells with additive manufacturing for high-efficiency water splitting. Appl. Energy.

[151] Yang G.Q., Yu S.L., Mo J.K., Kang Z.Y., Dohrmann Y., List F.A., Green J.B., Babu S.S., Zhang F.Y. (2018). Bipolar plate development with additive manufacturing and protective coating for durable and high-efficiency hydrogen production. J. Power Sources.

[152] Munsch M. (2017). Laser additive manufacturing of customized prosthetics and implants for biomedical applications. Laser Additive Manufacturing.

[153] Sun Y.X., Tian W., Zhang T., Chen P., Li M.J. (2020). Strength and toughness enhancement in 3d printing via bioinspired tool path. Mater. Des..

[154] Soundararajan R., Jayasuriya N., Vishnu R.G.G., Prassad B.G., Pradeep C. (2019). Appraisal of Mechanical and Tribological Properties on PA6-TiO_2_ Composites through Fused Deposition Modelling. Mater. Today Proc..

[155] Jonnala U.K., Sankineni R., Ravi Kumar Y. (2023). Design and development of fused deposition modeling (FDM) 3D-Printed Orthotic Insole by using gyroid structure. J. Mech. Behav. Biomed. Mater..

[156] Kumar R., Kumar M., Chohan J.S. (2021). The role of additive manufacturing for biomedical applications: A critical review. J. Manuf. Process.

[157] Masood S.H., Song W.Q. (2004). Development of new metal/polymer materials for rapid tooling using Fused deposition modelling. Mater. Des..

[158] Chohan J.S., Singh R. (2017). Pre and post processing techniques to improve surface characteristics of FDM parts: A state of art review and future applications. Rapid. Prototyp. J..

[159] Singh S., Singh G., Prakash C., Ramakrishna S. (2020). Current status and future directions of fused filament fabrication. J. Manuf. Process.

[160] Singh R., Davim J.P. (2018). Additive Manufacturing: Applications and Innovations.

